# Mild Cognitive Impairment with a High Risk of Progression to Alzheimer’s Disease Dementia (MCI-HR-AD): Effect of Souvenaid^®^ Treatment on Cognition and ^18^F-FDG PET Scans

**DOI:** 10.3233/ADR-190109

**Published:** 2019-05-03

**Authors:** Maria Sagrario Manzano Palomo, Belen Anaya Caravaca, Maria Angeles Balsa Bretón, Sergio Muñiz Castrillo, Asuncion de la Morena Vicente, Eduardo Castro Arce, María Teresa Alves Prez

**Affiliations:** a Department of Neurology, Infanta Leonor Hospital, Madrid, Spain; b Behavioral Neurology and Dementia Group of the Spanish Society of Neurology, Barcelona, Spain; c Neuropsychologist, Infanta Cristina Hospital, Parla, Madrid, Spain; d Department of Nuclear Medicine, Getafe Hospital, Madrid, Spain; e Department of Neurology, Infanta Cristina Hospital, Parla, Madrid, Spain; f Nutricia NeuroScience Medical Affairs, Madrid, Spain; g Nutricia Advanced Medical Nutrition, Madrid, Spain

**Keywords:** Alzheimer’s disease, ^18^F-FDG PET scans, mild cognitive impairment, neuropsychology, Souvenaid^®^

## Abstract

**Background:**

Previous studies have shown that Souvenaid (medical food) can have benefits on memory, cognition, and function in early Alzheimer’s disease (AD) and mild cognitive impairment (MCI).

**Objective:**

Demonstrate that Souvenaid could improve or maintain cognition and has an effect on neurodegeneration biomarkers.

**Methods:**

This cohort study was carried out from June 2015 through December 2016 in the Neurology Department, Infanta Cristina Hospital, Madrid, Spain. MCI-HR-AD were recruited using Petersen criteria, neuropsychology (NPS), and ^18^F-FDG PET scans to confirm the high risk of progression to dementia with one year of follow-up. Age, sex, vascular risk factors (VRF), and NPS values (Barcelona brief version) were analyzed. ^18^F-FDG PET scans were analyzed as a visual procedure. The study was approved by the Research Committee of ICH. Statistical analysis was made with SPSS 22.0 version.

**Results:**

Subjects included 43 MCI patients (58.5% women; mean age 69.78±7.89): 17 receiving Souvenaid^®^ treatment (ST), 24 receiving no treatment (WT) and 2 who withdrew. No differences were seen in VRF, only hypercholesterolemia, and were less prevalent in the ST group (*p* = 0.002). The rate of progression to dementia was 48.8% (no differences between groups, *p* = 0.654). A second round of ^18^F-FDG PET scans showed a significance worsening of glucose metabolism in WT (*p* = 0.001) versus ST, in which it was low (*p* = 0.050). For NPS testing, there was a significant worsening in memory performance in the WT group (*p* = 0.011) and a stabilization in ST (*p* = 0.083), as well as in executive functions and attention (worsening in WT, *p* = 0.014). For the Subjective Changing Scale (SCS), caregivers indicated a stabilization/improvement in ST (*p* = 0.017).

**Conclusion:**

Souvenaid had a significant effect on several cognitive domains, and on SCS in patients with MCI-HR-AD. Its intervention had an impact on preservation on ^18^F-FDG PET scans.

## INTRODUCTION

Alzheimer’s disease (AD) is a neurodegenerative condition which is highly prevalent in old age [1–4]. The World Health Organization (WHO) and Alzheimer’s Disease International (ADI) estimate that costs for care of older people will continue to increase and that the number of diagnosed dementia will reach 132 million patients [2, 3]. According to the Alzheimer’s Association, 13% of people over 65 suffer from this disease in developed countries, and this number is increasing in developing countries. AD has a significant socio-economic impact, which will lead to increased economic burden in healthcare systems all over the world [1–4].

AD has an insidious onset with episodic memory loss being one of the earliest reported symptoms. Progress toward effective therapies has been hampered because by the time cognitive symptoms emerge, significant pathological change has already taken place.

Aging is considered the principal risk factor for sporadic AD. Other potential risk factors include depression in midlife, low education level, obesity, hypertension in the midlife, dyslipidemia, metabolic syndrome, and diabetes [5–8].

The initial asymptomatic phase (preclinical AD) continues into a prodromal phase with mild, but noticeable, cognitive impairment but functional autonomy [9, 10], and eventual progression to dementia. This gradual progression creates a window of opportunity for pharmacological and non-pharmacological interventions in early disease stages.

Prevention trials have involved multimodal, non-pharmacological approaches including dietary intervention [11–14]. Diet is an important modifiable risk factor for dementia [15].

The LipiDiDiet group is a research consortium, which has studied the preclinical and clinical impact of nutrition in AD. This research contributed to the development of the medical food Souvenaid (Nutricia, Zoetermeer, the Netherlands). The results of the clinical trial in prodromal AD reported stabilization of cognition and function (Clinical Dementia Rating Scale Sum of Boxes, CDR-SB) and amelioration of hippocampal atrophy (magnetic resonance imaging, MRI) over a two-year period [16].

The active component of Souvenaid (Fortasyn Connect^®^) is a multinutrient combination, containing docosahexaenoic acid (DHA); eicosapentaenoic acid (EPA); uridine monophosphate; choline; vitamins B12, B6, C, E, and folic acid; phospholipids; and selenium [17]. These nutrients were selected based on their biological properties, involved in metabolic pathways (Kennedy and PEMP pathways), and specifically combined to enhance efficacy in phospholipid turn over and improvement of synaptic formation. In animal models, including transgenic AD mice, dietary intervention with this multinutrient combination has been shown to enhance phospholipid synthesis, to maintain white and gray matter integrity, to reduce the impact of amyloid-induced neurodegeneration and loss of functional connectivity, to increase numbers of hippocampal cholinergic synapses, and to improve cholinergic neurotransmission and hippocampus-dependent cognitive performance [17–26].

In two previous randomized clinical trials, Souvenaid improved memory performance in patients with mild AD, over 3 and 6 months, respectively [28, 29]. Furthermore, increased neurophysiological measures of synaptic activity, and enhanced functional connectivity in the brain [30] were reported in the longer study. Another study in patients with more advanced AD, who were on stable AD medication, showed no significant add-on effect of the multinutritional intervention [31].

Across four clinical trials, Souvenaid was well tolerated with a positive safety profile, alone and in combination with cholinesterase inhibitors and memantine. The results of the clinical trials of Souvenaid are consistent in their report of benefits in cognition and memory with better outcomes in earlier intervention [16]. The size effect of this nutritional intervention has been published recently [32]. Small to moderate effect sizes have been observed on primary outcome memory function in patients with mild AD in two separate randomized controlled trials [28, 29]. These effect sizes are clinically detectable and similar to those seen in cholinesterase inhibitors.

No effects were observed on the co-primary outcome Alzheimer’s Disease Assessment Scale—Cognitive Subscale (ADAS-cog), due to the lack of sensitivity of this outcome in very early AD patients.

To further investigate the presumed effect of this multinutrient combination on synaptic function, a Dutch double-blind randomized controlled parallel-group single-center study exploring the *Effect of this specific Nutritional Intervention on cerebral Glucose Metabolism in early Alzheimer*’*s disease* has been examined and designated as NL-ENIGMA (Dutch Trial Register NTR4718, http://www.trialregister.nl/trialreg/admin/rctview.asp?TC54718) [33].

In this present study, the mode of action of the specific multinutrient combination is further explored using ^18^F-fluoro-deoxyglucose (^18^F-FDG-PET) and neuropsychological tests in clinical practice.

The results of one trial using EEG suggest that Souvenaid preserves the organization of brain networks in patients with mild AD within 24 weeks, hypothetically counteracting the progressive network disruption over time in AD. This result strengthens the hypothesis that Souvenaid affects synaptic integrity and function [34].

In this work, we want to replicate the results obtained by Souvenaid in this trial preserving the organization of brain networks using FDG-PET, a technique that is a direct index for synapse function and density because the uptake of ^18^F-FDG is driven by synaptic terminals generating ATP for synthesis, release, and recycling of neurotransmitters, the maintenance of the normal resting potential, and the recovery from action potentials [35, 36]. We examine patients with MCI with high rate of progression to AD dementia.

## METHODS

43 patients from the Neurology Department of Infanta Cristina, Parla, Madrid, Spain Hospital, diagnosed with MCI according to Petersen criteria [37] were included. For diagnosis of MCI, impairment in one or more cognitive domains has to be present, based on clinical interpretation of performances on a neuropsychological test battery (Barcelona brief version battery) [38], whereas independency of functional abilities is preserved.

^18^F-FDG scans (w300 MBq, 90–110 min post injection) was considered positive when abnormal binding was seen in at least one cortical ROI (i.e., lateral temporal, frontal, posterior cingulate, precuneus, and parietal). Scans were divided into normal, mild (temporal low glucose metabolism (LGM) uni- or bilateral), medium (temporo-parietal LGM unilateral), and high (temporo-parietal LGM bilateral).

Souvenaid (specific multinutrient combination Fortasyn Connect^®^) intervention was offered to all patients. Those who refused were included in control group. Some patients in the prodromal phase of the disease were given cholinesterase inhibitors as a primary treatment. These patients were then given a combination of treatments (Souvenaid and cholinesterase inhibitors/memantine).

### Procedures

This longitudinal study, with a 1-year follow-up, was carried out from June 2015 to December 2016 in the Neurology Department of Infanta Cristina Hospital, Madrid, Spain.

Baseline demographic information, including age, sex, education, family history of AD, date of diagnosis MCI or dementia, and rate of progression to dementia, was recorded.

All patients had a neurological history, physical examination, neurological examination, neuropsychological test exploration, CT scans, blood samples (including total protein levels), and ^18^F-FDG-PET imaging. The imaging was conducted in the Department of Nuclear Medicine at the University Getafe Hospital.

Neuropsychological tests and ^18^F-FDG-PET imaging were conducted at the inclusion visit, approximately 8 months later, and checked again at the 1-year follow-up (dependent on the delay in clinical visit). On the last clinical visit, we included a neurological examination and the Subjective Changing Scale (SCS) completed by the caregiver.

### PET assessment and analyses

^18^F-FDG-PET scan was performed on a Siemens PET-CT scanner. Patients were in a fasting state for at least 6 h prior to scanning. An intravenous line was placed in an upper extremity 15 min prior to administration of approximately 185 MBq (5mCi) of ^18^F-FDG, dissolved in 5 mL of saline. After waiting 30 min at rest and without visual or auditory stimuli, the PET scan was initiated with a low-dose CT scan for attenuation correction of PET data and subsequent acquisition of PET, 1 bed of 10 min duration. The analysis was visual.

### Cognitive assessment

Cognitive measures were made according to the Barcelona battery (brief version) which included orientation, memory, language, executive functions, gnosis, and praxis domains. This battery was administered once at baseline and then repeated 8 months later. The results were divided in accordance with the Barcelona brief version rules, with the following cut-off: deficient (*p* < 5), low performance (p5–p20), medium performance (p25–p75), and high performance (*p* > 80). Patients included in high performance group were excluded from the study.

### Statistical analyses

A descriptive analysis was performed. Comparisons between the groups [Souvenaid treatment (ST) versus no treatment (WT)] were conducted using chi-square and/or Fisher’s exact tests for categorical variables, and Student *t*-test and Mann-Whitney test for continuous variables. McNemar’s test and Test of Marginal Homogeneity were used to determine differences during follow up.

In all analyses, differences of *p* < 0.05 were considered statistically significant. Analyses were performed using IBM SPSS Statistics for Windows, version 22.0.

Several potential covariates and possible intervention effect moderators were defined: Mini-Mental State Examination at screening, diagnosis of dementia, relevant medical events, relevant medication, coexisting diseases, all the demographic and other baseline variables, and product compliance.

### Ethical and legal considerations

The study 224/2017 was approved by the Research Committee of ICH in 2015.

## RESULTS

Between June 2015 to December 2016, 43 participants were included to the study: 17 receiving Souvenaid^®^ treatment (ST), 24 without treatment (WT), and 2 who withdrew. The main reasons for dropout were negative to intake. Two patients in the WT group refused ^18^F-FDG PET scans. Two patients refused neuropsychological (NPS) examination (one in each group). Mean age was 69.78 (±7.89 SD) years, and 58.5% of participants were women. Further baseline characteristics of study participants are shown in (Table 1).

**Table 1:** Baseline characteristics

Baseline characteristics	ST (*n* = 17)	WT (*n* = 24)	*p*
Age (y) Mean (±SD)	72.18 (±6.34)	68.08 (±8.54)	0.102
Sex *n* (%)	9 (52.9%) men	8 (33.3%) men	0.209
	8 (47.1%) women	16 (66.7%) women
Hypercholesterolemia *n* (%)	2 (14.3%)	15 (68.2%)	0.002
High blood pressure *n* (%)	9 (52.9%)	10 (41.7%)	0.476
Diabetes mellitus *n* (%)	2 (11.8%)	4 (16.7%)	0.512
Total proteins Mean (±SD)	7.023 (±0.54)	6.96 (±0.47)	0.722

ST, Souvenaid treatment; WT, without treatment; SD, standard deviation; n, number.

There were no differences in vascular risk factors (VRF), and hypercholesterolemia was less prevalent in the ST group (*p* = 0.002) (Fig. 2).

**Fig.1:**
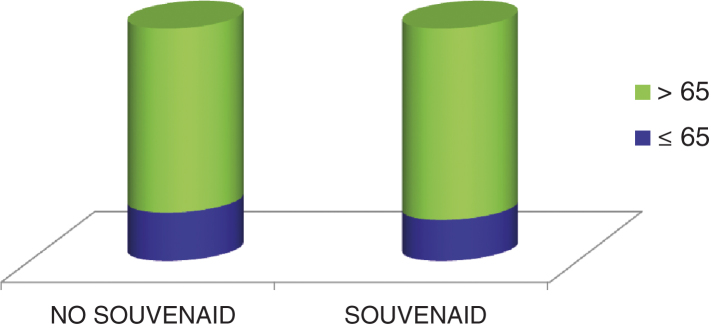
Age at onset.

The age of the participants at the beginning of the study was the same between groups (Fig. 1) and total protein was also the same in both groups (Fig. 3).

**Fig.2:**
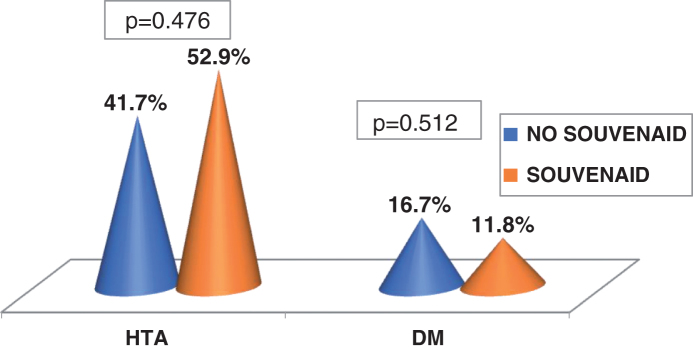
Cardiovascular risk factors. HTA, hypertension; DM, diabetes mellitus.

**Fig.3:**
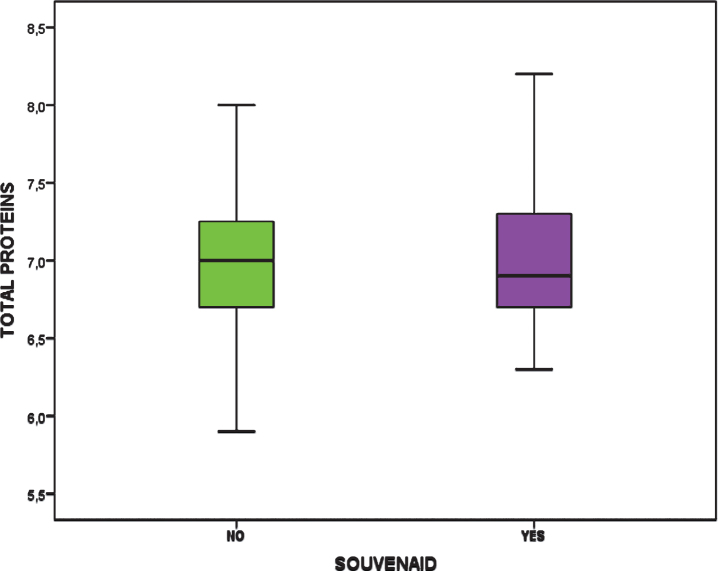
Total proteins in both groups.

In neuropsychological testing, each cognitive domain was evaluated. At baseline, a lower performance was observed in the ST group (*p* < 5 in 75% versus 34.8%, *p* = 0.033). There was a significant worsening in memory performance in the WT group (*p* = 0.011) from baseline, whereas stabilization was observed in the ST group (*p* = 0.083). Similarly for executive function and attention, the WT group experienced a worsening (*p* = 0.014) from baseline, while stabilization was seen in the in ST group.

Around 26% of the ST patients (*n* = 5) were receiving other treatment at the beginning (cholinesterase inhibitors, because of abnormal ^18^F-FDG PET scans or worse NPS performance). There were no differences between groups, comparing ST (monotherapy) with WT.

The rate of progression to dementia was higher in the bi-therapy group (ST monotherapy *p* < 0.008, WT *p* < 0.009), compared with monotherapy.

In the SCS, caregivers reported a stabilization or improvement in ST versus WT group (*p* = 0.017).

There were no differences in PET-FDG scans at baseline (*p* = 0.321). (See Table 2).

**Table 2:** ^18^F-FDG PET scans results (number and percentage)

	Normal	Mild impairment	Moderate impairment	Severe impairment	*p*
ST Group *n*(%)	*n* (%)	*n* (%)	*n* (%)	*n* (%)	0.050
First ^18^F-FDG PET scans	6 (35.29%)	8 (47.06%)	2 (11.76%)	1 (5.88%)
Second ^18^F-FDG PET scans	3 (17.65%)	8 (47.06%)	5 (29.41%)	1 (5.88%)
WT Group					0.001
First ^18^F-FDG PET scans	14 (63.64%)	8 (36.36%)	0 (0.00%)	0 (0.00%)
Second ^18^F-FDG PET scans	8 (36.36%)	7 (31.82%)	7 (31.82%)	0 (0.00%)

ST, Souvenaid treatment; WT, without treatment. *p*, McNemar’s test for ST group and test of marginal homogeneity for WT group.

At follow-up, ^18^F-FDG PET scans showed a significant worsening of glucose metabolism in WT (*p* = 0.001) versus ST, in which it was low (*p* = 0.050) (See Table 3).

**Table 3:** Main results. Comparison between groups

	WT	ST
PET	Significant worsening	Mild worsening
	*p* = 0.001	*p* = 0.050
Memory	Worsening	Stabilization
	*p* = 0.011	*p* = 0.053
Praxis	Stabilization	Stabilization
	*p* = 0.157	*p* = 1
Orientation	Stabilization	Stabilization
	*p* = 0.082	*p* = 0.999
Executive functions	Worsening	Stabilization
	*p* = 0.012	*p* = 0.135
Attention	Worsening	Stabilization
	*p* = 0.014	*p* = 0.157
Gnosis	Stabilization	Stabilization
	*p* = 0.151	*p* = 1

There were 3 patients with amyloid PET scans (one in ST and 2 in WT). In the ST patient, the SCS showed a mild improvement against the other two with a mild worsening.

There were no significant differences in the rate of progression to dementia between groups. The rate of progression to dementia was 48.8% (*p* = 0.654).

**Fig.4:**
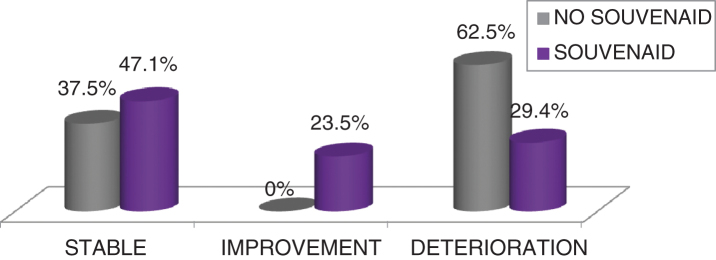
Subjective Changing Scale (SCS): differences between groups.

In the SCS, caregivers reported a stabilization or improving in ST versus WT (*p* = 0.017) (see Fig. 4).

## DISCUSSION

This study aims to explore the effect of Souvenaid on cerebral glucose metabolism in mild to very mild patients with high risk of progression to AD, using neuropsychological variables, and ^18^F-FDG scans over 1 year of follow up and in real life clinical practice. A positive effect of the nutritional intervention was observed, compared with controls, in cognitive and imaging parameters but not in progression to dementia. These results are in line with previous trials of Souvenaid in early AD [16].

This is the first Souvenaid study to include functional neuroimaging. ^18^F-FDG-PET is a well-established method to study synapse function [26–28].

The LipiDiDiet group, a research consortium, has studied preclinical and clinical impacts of nutrition in AD. This research contributed to the development of the medical food Souvenaid (Nutricia; Zoetermeer, the Netherlands). A clinical trial on the use of Souvenaid in prodromal AD reported that it stabilized cognition and function (CDR-SB) and ameliorated hippocampal atrophy (MRI) over a two-year period [16].

The active component of Souvenaid includes, in addition to other nutrients (e.g., B vitamins, vitamins C and E) [17], three food constituents which, when given together, promote synaptogenesis [19, 20]. These are choline, an omega-3 fatty acid [docosahexaenoic acid (DHA) or eicosopentaenoic acid (EPA)]; and uridine monophosphate (UMP). The omega-3 fatty acid and choline are true nutrients: their consumption in foods raises their levels in the blood and brain [20, 21]. In contrast, the uridine in the blood of adult humans is more like a hormone than a nutrient, in that it is derived not from dietary sources but from synthesis in and secretion from the liver [20, 21]. This is because most of the uridine in foods is present in a form (e.g., as RNA) which is not bioavailable in adult humans [22]. Infants do obtain dietary uridine from bioavailable sources, because much of the uridine in mothers’ milk or most infant formulas is present as UMP, the same bioavailable form as in Souvenaid [23].

These three compounds are essential precursors in the biosynthesis of the phosphatide molecules that comprise the bulk of synaptic membranes (phosphatidylcholine, phosphatidylethanolamine, phosphatidylserine, and phosphatidylinositol). The key biochemical steps in the conversions of DHA or EPA, choline, or uridine to the phosphatides are all catalyzed by enzymes which have low affinities for their substrates [20]. Thus, administering each substrate increases the saturation of its metabolizing enzyme and the rate at which new product is formed, ultimately raising brain phosphatide levels [19].

Administering the mixture of phosphatide precursors also increases brain levels of the major proteins in synaptic membranes, possibly via uridine’s activation of P2Y receptors [24]. Hence both of the key constituents (phospholipids and proteins) of functionally-complete membranes are formed when animals or humans receive the three precursors. This, in turn, enhances the production of dendritic spines [25], the immediate cytologic precursor of new synapses, thereby partly correcting the deficiency in the spines that is characteristic of AD and other dementias [26], and enabling enhanced synaptogenesis.

In animal models, including transgenic AD mice, administration of the three precursors, alone or as a constituent of Souvenaid, has also been shown to maintain the integrity of white and gray matter, reduce the loss of functional connectivity, increase cholinergic hippocampal synapses and cholinergic neurotransmission, and facilitate hippocampus-dependent cognitive performance [17–26].

A previous clinical study in patients with mild AD using EEG as a biomarker demonstrated an effect of the multinutrient combination on functional connectivity and brain network organization, suggesting that its mode of action includes alteration of synapse function [39].

As with all real-world studies, this has some limitations. The first one is the brief neuropsychological test battery used for the study, and the other is the visual assessment of ^18^F-FDG-PET scans.

Secondly, in the 5 patients with bi-therapy (ST and cholinesterase inhibitors), compared with the other (monotherapy or WT), there were no differences in the values, only in the rate of progression to dementia. Obviously, these patients were more impaired (although MCI criteria), therefore it was right to begin treatment sooner, with both therapies (ST and cholinesterase inhibitors). Interestingly, these patients progressed to dementia more quickly.

Another thing to consider is the option of to treat or not. We offered the treatment to everybody. All those who refused (we offered all the information about benefits and inconvenience) to take the treatment were included in the without treatment group. Another thing to consider is that the patients who withdrew from taking treatment did so for one reason, they did not want to drink a fluid. This is something to consider in the methodology of the study. They obviously stopped the follow up.

Overall, the results of this study support the hypothesis that Souvenaid, a multinutrient combination, can benefit patients with MCI and those who are at risk of progressing to AD. The NL-ENIGMA study will provide more information about this [33].

Souvenaid, as a medical food, had a significant effect on several cognitive domains in patients with AD in dementia stage and also in MCI. In our study, caregivers report benefits in the SCS in patients with MCI-HR-AD. Furthermore, its intervention had an impact on preservation as seen in ^18^F-FDG PET scans and in many cognitive domains (memory, attention, and executive functions).

## CONFLICT OF INTEREST

The authors have no conflict of interest to report.
